# Rare and unusual case of anti-factor XI antibodies in patient with plasma cell leukemia

**DOI:** 10.1186/s12878-018-0100-9

**Published:** 2018-08-10

**Authors:** Jean Uwingabiye, Hafid Zahid, Mohamed El Amrani, Fayçal Labrini, Abdelhak Elkhazraji, Driss El Kabbaj, Mohammed Benyahia, Anass Yahyaoui, Rachid Hadef, Nezha Messaoudi

**Affiliations:** 1 Laboratory of Hematology and Immunohematology, Mohammed V Military Teaching Hospital, Faculty of Medicine and Pharmacy, Mohammed V University, Rabat, Morocco; 2 Nephrology Department, Mohammed V Military Teaching Hospital, Faculty of Medicine and Pharmacy, Mohammed V University, Rabat, Morocco

**Keywords:** Activated partial thromboplastin time, Russell’s viper venom time, Blood coagulation factor inhibitors, Monoclonal gammopathy, Plasma cell leukemia

## Abstract

**Background:**

The acquired inhibitors of coagulation have been observed in very rare cases of monoclonal gammopathies. We report a very rare case of anti-factor XI antibodies in patient with plasma cell leukemia (PCL).

**Case presentation:**

This is a 59-year-old male patient without pathological history, admitted to the nephrology department for management of renal insufficiency and anemia syndrome. The history and physical examination revealed stigmata of hemorrhagic syndrome including hemothorax and hemoptysis. The hemostasis assessment showed an isolated prolonged activated partial thromboplastin time (APTT) with APTT ratio = 2.0.The index of circulating anticoagulant (37.2%) revealed the presence of circulating anticoagulants. The normalized dilute Russell viper venom time ratio of 0.99 has highlighted the absence of lupus anticoagulants. The coagulation factors assay objectified the decrease of the factor XI activity corrected by the addition of the control plasma confirming the presence of anti-factor XI autoantibodies. In addition, the blood count showed bicytopenia with non-regenerative normocytic normochromic anemia and thrombocytopenia. The blood smear demonstrated a plasma cell count of 49% (2842/mm3) evoking PCL. The bone marrow was invaded up to 90% by dystrophic plasma cells. The biochemical assessment suggested downstream renal and electrolyte disturbances from exuberant light chain production with abnormalities including hyperuricemia, hypercalcemia, elevated lactate dehydrogenase, non nephrotic-range proteinuria and high level of C reactive protein. The serum protein electrophoresis showed the presence of a monoclonal peak. The serum immunofixation test detects the presence of monoclonal free lambda light chains. He was treated with velcade, thalidomide and dexamethasone. The patient died after 2 weeks despite treatment.

**Conclusion:**

Both PCL and anti-factor XI inhibitors are two very rare entities. To the best of our knowledge, this is the first reported case of a factor XI inhibitor arising in the setting of PCL. Factor inhibitors should be suspected in patients whose monoclonal gammopathies are accompanied by bleeding manifestations.

## Background

Patients with monoclonal gammopathies may have hemostasis disorders with a double risk: bleeding and thrombosis risks. The bleeding risk is generally associated with the secreted immunoglobulin (Ig) responsible for hyperviscosity syndrome, thrombopathy by binding Ig to platelets, autoantibodies to coagulation factors, existence of thrombocytopenia and treatment whereas the thrombotic risk may be linked to paraneoplastic phenomena or to treatment such as thalidomide derivatives and dexamethasone [1, 2].

The anti-factor XI autoantibodies are very rare and have been reported in some monoclonal gammopathies such as Waldenström macroglobulinemia [3] and in other malignant hemopathies such as chronic lymphocytic leukemia and chronic myeloperocytic leukemia [4] . They have also been found in: autoimmune diseases, lung cancers, prostate adenocarcinoma, heart disease, liver disease, dermatological disorders and in viral infections. These antibodies may also appear in patients with deficiency after repeated infusions of fresh frozen plasma, antibiotic therapy, chlorpromazine or procainamide therapy [4]. To our knowledge, no case of anti-factor XI antibodies in a patient with plasma cell leukemia (PCL) has been described in the literature. We report a very rare case of anti-factor XI antibodies in patient with plasma cell leukemia (PCL).

## Case presentation

This is a 59- year-old -male patient without pathological history, followed in the nephrology department of the Mohammed V Military Teaching Hospital for renal insufficiency and anemia syndrome. The history and physical examination revealed stigmata of hemorrhagic syndrome including hemothorax and hemoptysis. The patients was not treat with anticoagulants.

The hemostasis assessment showed an isolated prolonged activated partial thromboplastin time (APTT) with APTT ratio of 2.0 (normal < 1.2). The prothrombin time (PT) (87%), the bleeding time (2 min and 30 s) and the fibrinogen level (2.88 g/l) were in the range of their physiologic values.

The exploration of prolonged APTT included: the confirmation of the prolongation of the APTT on two successive samples by using two different reagents: STA®-Cephascreen® (Diagnostica Stago) and STA®-PTT® automaton (Diagnostica Stago).The correction of the APTT in the mixing study performed by mixing equal parts of the patient’s plasma with normal pooled plasma, demonstrated the presence of circulating anticoagulants,the index of circulating anticoagulants was 10.7% and 37.2%, respectively, before and after 2 h incubation at 37 °C (normal < 15%), the dilute Russell viper venom time (dRVVT) showed the absence of lupus anticoagulants (LA) antibodies with normalized ratio of 0.99 (normal< 1.20) and the intrinsic pathway factors assay objectified the decrease of the factor XI activity corrected by the addition of the control plasma confirming the presence of anti-factor XI autoantibodies (Table 1).

**Table 1:** Hematologic assessment of patient

Parameters	Patients		Reference values
Blood count			
White blood cells (10^3^/μl)	5.8		4–11
Red blood cells (10^6^/μl)	2.47		4.5–5.7
Hemoglobin (g/dl)	7.1		13–17
Mean corpuscular volume (fl)	83		80–100
Mean Corpuscular Hemoglobin (pg)	28.9		27–32
Mean Corpuscular Hemoglobin Concentration (%)	34.9		32–36
Absolute reticulocyte count (ARC) (/μl)	60,800		*
Platelets (10^3^/μl)	22		150–450
Blood smear			
Circulating plasma cells (%)	49		0
Bone marrow smear			
Bone marrow plasma cells (%)	90		0–2
Hemostasis assessment			
First-line screening tests for hemostasis			
APTT (seconds)	72		36…44
Prothrombin time (%)	87		70–100
Fibrinogen levels (g/L)	2.88		2–4
Bleeding Time - 3 points Ivy Method (minutes)	2.5		2–4
Exploration of prolonged APTT			
Index of circulating anticoagulants			
Without incubation			
APTT Patient (seconds)	70.8		33….41
APTT Mixed (Patient + Normal) (seconds)	40.6		33….41
APTT Normal (seconds)	33.0		33….41
Index of circulating anticoagulants (%)	10.7		> 15
After 2 h incubation at 37 °C.			
APTT Patient (seconds)	110.5		33….41
APTT Mixed (Patient + Normal) (seconds)	85.5		33….41
APTT Normal (seconds)	44.3		33….41
Index of circulating anticoagulants (%)	37.2		> 15
Diluted Russell Viper Venom time			
Normalized ratio: (DRVV screen ratio)/ (DRVV confirm ratio)	0.99		< 1.20
Determination of coagulation factors activities	Patient	Patient + Normal	Reference values
Factor II	61%	79%	70….120%
Factor IX	175%	127%	60….150%
Factor X	74%	64%	70…..120%
Factor XII	102%	123%	60….150%
Factor VIII	728%	379%	60….150%
VWF: Ag	438%	344%	50…..160%
Factor XI	13%	10%	60….150%

*=ARC< 120,000/μl: non-regenerative anemia and ARC> 120,000/μl: regenerative anemia APTT = Activated partial thromboplastin time, VWF: Ag = VWF: Von willebrand factor antigen, DRVVT: dilute Russell viper venom time, Patient +Normal: mixing the patient’s plasma in equal parts with a pool of normal plasma

Furthermore, the blood count showed bicytopenia with non-regenerative normocytic normochromic anemia (Hemoglobin = 7.1 g/dL, mean corpuscular volume = 83 fl, mean corpuscular hemoglobin = 28.9 pg, mean corpuscular hemoglobin concentration = 34.9%, absolute reticulocyte count: 60800 / μl) and thrombocytopenia at 22000/μl. The blood smear demonstrated a plasma cell count of 49%, or 2.842 Giga / l evoking PCL. The bone marrow examination showed marrow invaded up to 90% by dystrophic plasma cells.

The biochemical assessment suggested downstream renal (hypercreatininaemia at 27.7 mg/dl and kidney failure with glomerular filtration rate estimated according to Modification of Diet in Renal Disease (MDRD) = 2 ml/min/1.73 m^2^) and electrolyte disturbances from exuberant light chain production with abnormalities including hyperuricemia (15 mg/dl), hypercalcemia (110 mg/l), elevated lactate dehydrogenase (LDH) (943UI/l), non nephrotic-range proteinuria (1.26 g/24 h) and high level of C reactive protein (CRP) (13.2 mg/l) .

The serum proteins electrophoresis revealed the presence of monoclonal peak migrating in the beta-1 globulins region amounted to 9.92 g/l with significant hypogammaglobulinemia. The immunofixation showed the presence of monoclonal free lambda light chains.

The radiological assessment did not show bone damage.

The patient was dialyzed, transfused and then treated with velcade, thalidomide and dexamethasone. He died after 2 weeks due to respiratory distress secondary to alveolar hemorrhage.

## Discussion and conclusion

The coagulation abnormalities may be encountered in patients with monoclonal gammopathies. In a Japanese study, 14% of patients with multiple myeloma (MM) had prolonged APTT [5] whereas in American study, prolonged PT, prolonged APTT and prolonged thrombin time (TT) were observed in 59%, 18% and 71% of patients with MM respectively [6]. In all monoclonal gammopathies, haemorrhagic manifestations may be due to abnormal thrombocytopenia and / or to platelet function, to the inhibitory effect of Ig on certain coagulation factors or to binding of circulating Ig to coagulation factors leading to rapid clearance of antigen complexes, and hyperviscosity syndrome [4]. Previous studies have reported some cases of association of acquired inhibitors of coagulation with monoclonal gammopathies [3, 7–11]. Inhibitory antibodies against coagulation factors are acquired pathological inhibitors of coagulation. They are divided into two sub-groups of unequal importance and different clinical expression: the first consists of antibodies directed against a single coagulation factor which generally expose to hemorrhagic accidents. The second group includes antibodies directed against a phase of coagulation which expose rather to accidents of thrombosis [12] .The acquired factor VIII inhibitors has been reported in 4 cases of MM with hemorrhagic manifestations; 2 men and 2 women, aged 43 to 70 years with a mean age of 57.25 ± 11 years, presenting hemorrhagic manifestations and two of these patients died [7–10](Table 2).The coexistence of MM and the acquired coagulation inhibitor to factor II was found in a 52-year-old patient with hemorrhage and whose clinical course was complicated by death [11] (Table 2).The fibrino formation inhibitors were observed in two cases of myeloma; in the first one, these inhibitors were responsible for severe bleeding that contributed to the death of the patient and in the second case of IgD myeloma with hypercalcemia, the inhibitors disappeared with the first chemotherapy course [12].The Willebrand factor Inhibitors were also found in 6 cases of monoclonal gammopathies of undetermined significance, 4 cases of Waldenstrom macroglobulinemia and 1 case of MM [13] (Table 2).

**Table 2:** Association of acquired coagulation factor inhibitors and monoclonal gammopathy: literary analysis

Authors (Year of publication)	Acquired coagulation factor inhibitors	Monoclonal gammopathy types	Age (years)	sex	Type of hemorrhage	Treatment	Clinical evolution
Sari (2009) [7]	anti-factor VIII antibody	Multiple myeloma	43	F	Postoperative	VAD (vincristine, doxorubicin and dexamethasone)	favorable
Glueck (1965) [8]	anti-factor VIII antibody	Multiple myeloma	70	M	cutaneous and retinal	cyclophosphamide	–
Hoyer (1995) [9]	anti-factor VIII antibody	Multiple myeloma	58	F	Soft tissue, gastrointestinal	Porcine factor VIII, activated prothrombin complex concentrate; Corticosteroids, plasma exchange	deceased
Holme (2005) [10]	anti-factor VIII antibody	Multiple myeloma	58	M	Postoperative	Activated prothrombin complex concentrate; Corticosteroids, cyclophosphamide	deceased
Cowell (1996) [11]	anti-factor II antibody	Multiple myeloma	52	M	hematuria, Intracranial hemorrhage, epistaxis	Plasmapheresis, melphalan, prednisone, Cyclophosphamide, transplantation	deceased
Godeau (1985) [12]	Acquired inhibitors of fibrin formation	2 cases of Multiple myeloma					
Kumar (2002) [13]	Anti-von Willebrand factor antibody	6 cases of monoclonal gammopathies of undetermined significance, 4 cases of Waldenström’s macroglobulinemia and 1 case of Multiple myeloma					
Reece (1984) [3]	anti-factor XI antibody	3 patients with Waldenström’s macroglobulinemia					

F: female, M: male

The anti-factor XI inhibitors have been reported in three patients with Waldenstrom macroglobulinemia [4], but no case of anti-factor XI inhibitors with PCL has been reported.

The acquired inhibitors of clotting factors are fast acting G or M immunoglobulins. They don’t have antiphospholipid activity. Their presence in plasma is characterized by prolonged APTT. PT and TT are normal. The prolonged APTT is not corrected with the normal plasma [4].

The APTT corresponds to the time required for clot formation of citrated platelet-poor plasma in the presence of phospholipids, calcium and contact activator. APTT explores the endogenous pathway. The prolonged APTT is defined as the ratio of patient’s APTT / control’s APTT greater than 1.2. In the case of the isolated Prolonged APTT, the assessment is completed by the realization of the correction of the APTT in the mixing study, the index of circulating anticoagulants formerly known as the Rosner Index, the search of circulating anticoagulants and the endogenous pathway factor (VIII, IX, XI, XII) analysis. The correction of the APTT in the mixing study is performed by mixing the patient’s plasma with control normal plasma for 2 h of incubation at 37 °C.

The Correction or non-correction of APTT is objectified by the calculation of the index of circulating anticoagulants which corresponds to the following formula:

Index of circulating anticoagulants = [(APTT mixed - APTT normal plasma) / APTT patient] × 100.

APTT is considered to be corrected if the index of circulating anticoagulants is less than 12%, the correction of APTT suggests coagulation factor deficiency and orientates towards the factors measurement. Any index of circulating anticoagulants value greater than 15% indicates the non-correction of the APTT and it is in favor of the presence of the circulating anticoagulants. When the index of circulating anticoagulants value is between 12% and 15%, the test is doubtful [13, 14].

The DRVVT allows to exclude the presence circulating lupus anticoagulant. The Russel viper venom converts factor X into an activated factor X in the presence of calcium ions. The activated factor x then acts to convert prothrombin to thrombin in the presence of activated factor V, calcium ions and phospholipid. It has the advantage of not being influenced by deficiencies in upstream of factor X, VII, VIII, IX or contact activation system of coagulation. It is rendered insensitive to heparin up to a concentration of 1 U/ml. The DRVVT sensitivity varies depending on the reagent used. This test makes it possible to confirm phospholipid-dependent coagulation inhibitor by correcting the prolongation of the tests after increasing the phospholipid concentration of the reagents, thereby neutralizing the activity of the lupus anticoagulants. The correction of the prolongation of the tests after increasing the phospholipid concentration of the reagents, which neutralizes the activity of the lupus anticoagulants. For confirmatory testing, it is recommended to express their result as a normalized ratio: (DRVVT patient screen/ DRVVT patient confirm) / (DRVVT screen control / DRVVT control confirm). The normalized ratio greater than 1.2 confirming the presence of lupus anticoagulants while the ratio less than 1.2 indicates the presence of acquired inhibitors of coagulation. There are other lupus anticoagulant screening tests such as diluted thrombin time and kaolin clotting time but their use is no longer recommended in this indication due to the lack of specificity [14, 15]. In our case, two of four International Society on Thrombosis and Hemostasis (ISTH) criteria for lupus anticoagulant [16] were met: prolongation of at least one of phospholipid-dependent clotting assay (isolated prolongation of the APTT in this case), and evidence of inhibitory activity in a mixing study using pooled normal plasma. But these two criteria are the same as those used for detection of blood coagulation factor inhibitors. The third ISTH criteria was not met as DRVVT showed the absence of the lupus anticoagulants. The fourth ISTH criteria was not completely met: there is exclusion of anticoagulant drugs but specific factor inhibitor was indeed determined. These results reasonably exclude lupus anticoagulant as a competing cause for APTT prolongation and non-correcting APTT mixing study. It’s clear that the acquired inhibitors of clotting factors were the cause of this hemostasis disorder.

In our case report, the levels of coagulation factors in intrinsic pathway were normal except factor XI activity which was decreased. The search for anti-factor XI inhibitors was carried out after incubation of the mixture of patient’s inhibitor plasma and normal plasma for 2 h at 37 °C in order to release the antibody from the factor XI-anti-factor XI antibody complex and determining residual clotting factor XI. In some specialized laboratories, the detection of this antibodies can be completed by the titration of the anti-factor XI by adding different dilutions of patient’s plasma (1/2 to 1 / 128th) to the control plasma. The percentage of residual factor XI activity is determined at each dilution. The anti-factor XI level is calculated based on the percentage of residual factor XI in the Besthesda unit, the 50% residual factor XI activity corresponds to a 1 Bethesda / ml unit [4].

Factor XI is a coagulation dimeric glycoprotein synthesized by the liver and it is part of the contact activation pathway which initiates the intrinsic coagulation pathway with factor XII, prekallikrein and high-molecular-weight kininogen (HMWK). It is activated by the activated factor XII and the thrombin to factor XIa which will itself activate the factor XI in the presence of calcium ions. This factor has the ability to activate thrombin-activable-fibrinolysis factor (inhibitor of fibrinolysis) and promotes fibrin formation by enhancing the stability of the clot and increasing its resistance to fibrinolysis. Therefore, anti-factor XI antibodies may be associated with decreases in other factors of the endogenous pathway including factor XII, prekallikrein, HMWK, factor VIII and factor IX, or may be associated with lupus anticoagulants. The combinations of inhibitors directed against both factor XI and factor XII have also been reported, so it is necessary to ensure the absence of the factor XIIa blocking antibody in the case of persistence of factor XII deficiency [4].

In this case report, the patient presented abnormal bleeding including hemoptysis and hemothorax. The clinical picture of these inhibitors, is variable, often parallel to the course of the underlying disease. These inhibitors are exceptionally responsible for hemorrhages and there is no correlation between bleeding manifestations and the factor XI level and the inhibitor titer. Hemorrhage rarely occurs during trauma or surgery. They often concern tissues rich in fibrinolysis activators such as the otorhinolaryngology sphere or the urinary tract. The use of invasive procedures can lead to severe hemorrhagic. Bleeding disorders such as abdominal bleeding due to a ruptured ovarian cyst, excessive bleeding following transurethral resection of the prostate recurrent fetal loss, renal bleeding after trauma, retroperitoneal bleeding, spontaneous ecchymoses, subarachnoid hemorrhage, gastrointestinal bleeding, hematochezia and hemoptysis have been reported in the previous studies in patients with factor XI inhibitors. If these inhibitors are associated with lupus anticoagulants, severe factor XII deficiency (< 1%) or anti-factor XII antibody, the stigmata of coagulopathies including the occurrence recurrent thrombosis, finger thrombosis and ocular vessel thrombosis, can be occurred. These inhibitors may persist throughout life or disappear after six months to a year of evolution. Regular biological and clinical monitoring to monitor the progress of the inhibitor is necessary [3, 4]. Therapeutic management should be discussed in relation to the occurrence of hemorrhagic syndrome, not to the plasma level of factor XI.

The treatment involves corticosteroids, high-dose immunoglobulins, immunosuppressants (Imurel®, Endoxan®), plasmapheresis or massive doses of coagulant factor that can neutralize the inhibitor. In particular cases of association of cancer inhibitors, chemotherapy should be proposed, and may be associated with corticosteroids [4].

**Fig. 1:**
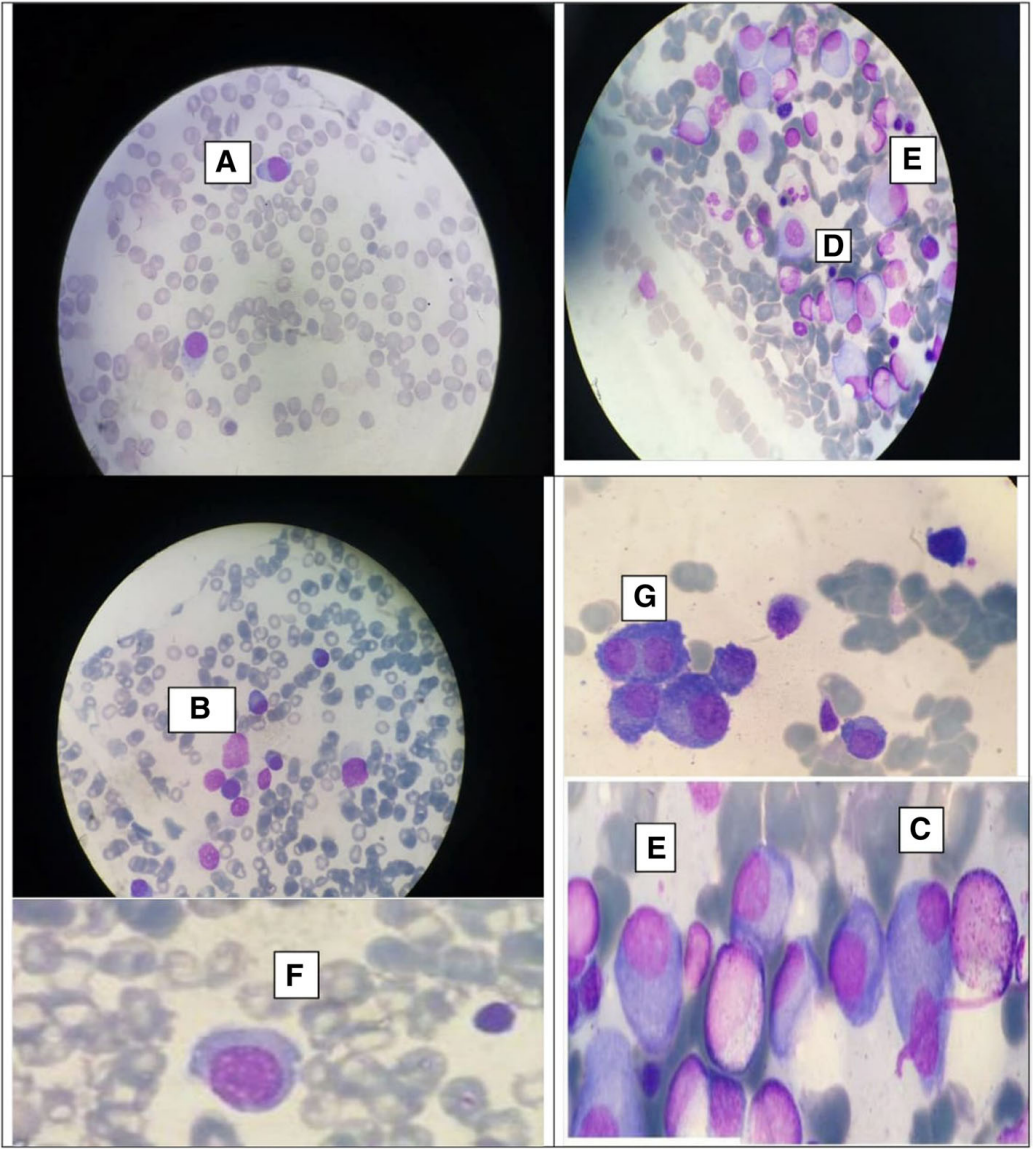
Blood and medullary smear showing plasma cells: (**a**) normal plasma cells in the peripheral blood smear, (**b**) normal plasma cells in the bone marrow, dystrophic plasma cells in the bone marrow ((**c**) Binucleated plasma cells, (**d**) Plasma cells with high nucleo-cytoplasmic ratio, (**e**) large plasma cells, (**f**) Flaming Plasma Cells, (**g**) plasma cells nests (May-Grünwald-Giemsa staining, magnification: X1000)

The diagnosis of plasma-cell leukemia was based on the detection of plasma more than 20% plasma cells in the peripheral blood and on an absolute count greater than 2 Giga / L plasma cells. The circulating plasma cells morphology was closely related to that of the medullary plasma cells: very basophilic cytoplasm and small oval nucleus eccentric in the cell (Fig. 1).The dystrophic plasma cells were also often observed (Fig. 1). Previous studies reported that during this pathology, the bone marrow is clearly infiltrated with plasma cells with mean values ranging from 76 to 83% [17]. Some researchers established a Plasma cell labeling index (percentage of S-phase plasma cells after in vitro bromodeoxyuridine incorporation) and have shown that the plasma-cell clone is much more proliferating in PCL than in MM [17, 18]. PCL is a particular form of aggressive monoclonal gammopathy and represent 1 to 2% of malignant hemopathies [19]. There are two forms of PCL: primary PCL occurring de novo in patients without preexisting MM and secondary PCL corresponding to a late event found in 1% of patients with MM [19]. In our case, the patient presented a primary PCL. The anemia and thrombocytopenia found in our patient are the most frequent biological abnormalities in PCL; anemia is usually normocytic, normochromic with less than 10 g /dL in 45 to 87.5% of patients, platelet count is less than 10^5^/μl in 50% of patients with PCL and is higher in patients with primary PCL than in patients with secondary PCL [20]. These cytopenias are secondary to the suppressive effect on the myeloid progenitors of cytokines released by the natural killer cells present in the marrow of the PCL patients. Interferon-like cytokines released by plasma cells could also have a suppressive effect [21]. In addition to renal insufficiency, our patient also presented hypercalcemia and high LDH levels which are known as the poor prognosis factors in this disease [22]. C-reactive protein was also increased, reflecting the amount of circulating interleukin-6 and it is increased in 61% of PCL. Most patients with PCL have a serum or urinary monoclonal peak but non-secreting PCL cases have been reported [19]. The PCL is more frequently responsible for extramedullary manifestations compared to MM [17, 18]. Extramedullary infiltration by plasma cell leads to organomegaly including hepatomegaly, splenomegaly, lymphadenopathy and leptomeningeal infiltration and lytic bone lesions. Our patient had no signs of organomegaly or those of bone lesions [18, 23]. The PCL is usually very poor prognosis; the median survival of PCL from treatment initiation varies from 6.8 to 12.6 months [17, 18] and about 28% of patients die within the first month of diagnosis [24]. Treatment of PCL by alkylating agents such as melphalan in combination with glucocorticoids is not effective [25]. Variable multidrug therapy such as VAD (vincristine, doxorubicin, dexamethasone), modified VAD with liposomal doxorubicin, VAMP (vincristine, doxorubicin, methyl prednisolone), VBAP (vincristine, BCNU, doxorubicin, prednisone),VMD (vincristine, mitoxantrone, Dexamethasone), CVP (cyclophosphamide, vincristine, prednisone), and VMBCP (vincristine, melphalan, BCNU, cyclophosphamide, prednisone) showed response rates varying from 50 to 75% [20]. Compared with MM, PCL are more chemoresistant because of its biological characteristics related to the intrinsic malignancy of the disease: frequency of adverse cytogenetic abnormalities (hypodiploidy, deletion of chromosome 13), low index DNA and more immature phenotype [20]. Given the poor prognosis of PCL, innovative therapies such as thalidomide and its analogues (CC5013), proteasome inhibitors (bortezomib), and bone marrow allograft with attenuated conditioning regimen have been evaluated. Bortezomib, a proteasome inhibitor, showed good activity against primary and secondary PCL. Thalidomide has shown a significant interest in the treatment of PCL even though there is no evaluation of this therapy in the literature [20, 23]. Lenalidomide has been found to be less toxic and more effective than thalidomide. Lenalidomide in combination with dexamethasone appear to be effective with or without autologous or allogeneic stem cell transplantation. Novel drug combination therapy including bortezomib, thalidomide, and dexamethasone (VTD),lenalidomide, bortezomib, and dexamethasone (RVD) or melphalan, prednisone, bortezomib, and thalidomide (VMPT) provided the very promising results but their efficacy were based on small numbers of patients. The efficacy of tandem autologous and allogeneic stem cell transplantation remains to be proved [18, 23].In our case, the patient was treated with velcade thalidomide and dexamethasone and died after 2 weeks due to respiratory distress secondary to alveolar hemorrhage.

Both PCL and anti-factor XI inhibitors are very rare. This case confirms that PCL remains aggressive hemopathy and poor prognosis. To the best of our knowledge, this is the first reported case of a factor XI inhibitor arising in the setting of PCL. Factor inhibitors should be suspected in patients whose monoclonal gammopathies are accompanied by bleeding manifestations. The anti-factor XI inhibitors is highlighted by isolated prolonged APTT not corrected by the addition of normal plasma and the decrease of the factor XI activity corrected by the addition of normal plasma. Anti-factor XI inhibitors are rarely responsible for hemorrhagic syndrome, but their clinical course is often parallel to the course of the underlying disease. Therefore, regular biological and clinical monitoring to monitor the progress of the inhibitor is necessary. Further studies are needed to achieve a better understanding of the occurrence of anti-factor XI inhibitors in patients with PCL.

## Abbreviations

APTT: activated partial thromboplastin time
ARC: Absolute reticulocyte count
CRP: C reactive protein
CVP: cyclophosphamide, vincristine, prednisone
dRVVT: dilute Russell viper venom time
HMWK: high-molecular-weight kininogen
Ig: immunoglobulin
ISTH: International Society on Thrombosis and Hemostasis
LA: lupus anticoagulants
LDH: lactate dehydrogenase
LDH: lactate dehydrogenase
MDRD: Modification of Diet in Renal Disease
MM: multiple myeloma
PCL: plasma cell leukemia
PT: prothrombin time
RVD: lenalidomide, bortezomib, and dexamethasone
TT: thrombin time
VAD: vincristine, doxorubicin, dexamethasone
VAMP: vincristine, doxorubicin, methyl prednisolone
VBAP: vincristine, BCNU, doxorubicin, prednisone
VMBCP: vincristine, melphalan, BCNU, cyclophosphamide, prednisone
VMD: vincristine, mitoxantrone, Dexamethasone
VMPT: melphalan, prednisone, bortezomib, and thalidomide
VTD: bortezomib, thalidomide, and dexamethasone
